# Efficacy of nano-hydroxyapatite on caries prevention—a systematic review and meta-analysis

**DOI:** 10.1007/s00784-022-04390-4

**Published:** 2022-02-01

**Authors:** Richard Johannes Wierichs, Thomas G. Wolf, Guglielmo Campus, Thiago S. Carvalho

**Affiliations:** 1 Department of Restorative, Preventive and Pediatric Dentistry, School of Dental Medicine, University of Bern, 3010 Bern, Switzerland; 2 Department of Periodontology and Operative Dentistry, University Medical Center of the Johannes Gutenberg-University Mainz, 55131 Mainz, Germany; 3 Department of Surgery, Microsurgery and Medicine Sciences, School of Dentistry, University of Sassari, Viale San Pietro 3/c, 07100 Sassari, Italy; 4 Faculty of Dentistry, Sechenov First Moscow State Medical University, Moscow, 119991 Russia

**Keywords:** Dental caries, Meta-analysis, Systematic review, Microinvasive treatment, Nano-hydroyapatite, Micro-hydroyapatite, White spot lesions

## Abstract

**Introduction/objectives:**

The review systematically explored in vivo or in situ studies investigating the efficacy of nano-hydroxyapatite (nHA) to reduce initiation of or to remineralize initial caries lesions.

**Data:**

Prospective controlled (non-)randomized clinical trials investigating the efficacy of a nHA compared to any other (placebo) treatment or untreated/standard control.

**Sources:**

Three electronic databases (Central Cochrane, PubMed-MEDLINE, Ovid EMBASE) were screened. Outcomes were, e.g., ICDAS score, laser fluorescence, enamel remineralization rate, mineral loss, and lesion depth. No language or time restrictions were applied. Risk of bias and level of evidence were graded using the Risk of Bias 2.0 tool and GRADE profiler.

**Study selection/results:**

Five in vivo (and 5 in situ) studies with at least 633 teeth (1031 specimens) being assessed in more than 420 (95) patients were included. No meta-analysis could be performed for in vivo studies due to the high heterogeneity of the study designs and the variety of outcomes. In situ studies indicate that under demineralization conditions, NaF was able to hinder demineralization, whereas nHA did not; simultaneously, nHA did not differ from the fluoride-free control. In contrast, under remineralizing conditions, nHA and NaF show the same remineralizing potential. However, the level of evidence was very low. Furthermore, six studies showed a high risk of bias, and six studies were funded/published by the manufacturers of the tested products.

**Conclusion:**

The low number of clinical studies, the relatively short follow-up periods, the high risks of bias, and the limiting grade of evidence do not allow for conclusive evidence on the efficacy of nHA.

**Clinical relevance:**

No conclusive evidence on the efficacy of nHA could be obtained based on the low number of clinical studies, the relatively short follow-up periods, the high risks of bias, the limiting grade of evidence, and study conditions that do not reflect the everyday conditions.

**Supplementary Information:**

The online version contains supplementary material available at 10.1007/s00784-022-04390-4.

## Introduction

In recent years, a trend towards lower caries prevalence could be observed due to higher efforts in caries prevention [1, 2]. However, initial caries can still be frequently observed. These lesions have higher pore volume and decreased hydroxyapatite volume [3]. Consequently, light is scattered differently compared with sound enamel, and the lesions appear opaque. Thus, they were often called white spots or more recently initial caries lesions [4].

To enhance the remineralization of the initial caries lesions, application of fluoride-containing agents [5] and casein phosphopeptide-amorphous calcium phosphate (CPP-APP) containing pastes [6], bioactive glasses [7], or self-assembling peptides [8] may be used. Furthermore, synthetic nano-hydroxyapatite (Ca_10_(PO_4_)_3_(OH)_2_)/zinc-carbonate-hydroxyapatite nanocrystals (nHA) is a bioactive-compatible material with similar chemical composition to the apatite crystals of human enamel, and it has also been used as micro-cluster or nanocrystalline forms to induce remineralization [9, 10]. Based on its ability to strongly adsorb to tooth surfaces, nHA has shown the in vitro capacity to fill enamel interprismatic spaces and thus possibly remineralize the enamel. Several experiments have compared nHA-containing dentifrices to positive (fluoride-containing) or negative control dentifrices, resulting either in net-remineralizing or net-demineralizing conditions. Net-remineralizing condition is when even the (negative) control groups remineralize, not necessarily related to the dentifrices but due to the experimental cycling model itself, whereas a “net-demineralizing” condition is when even the positive control groups demineralize. In studies that resulted in net-remineralization model, a nHA-containing dentifrice (10 wt%) caused an enamel mineral gain, which was not significantly different compared with an amine fluoride dentifrice (1450 ppm F^−^) [9]. Also, no significant difference in mineral gain was observed between a nHA (10 wt%) and a NaF-containing dentifrice (1100 ppm F^−^) in situ [10]. Contrastingly, under net-demineralizing in vitro conditions, negative results have been observed [11, 12], where enamel subsurface demineralization occurred with the experimental pastes containing 10% or 20% nHA, and no differences were observed to the placebo treatment (paste without nHA and F^−^) or to the no-treatment groups [11]. Furthermore, the dentifrice containing nHA produced comparable results to the negative control (paste without nHA and F^−^) even when other fluoride dentifrices were able to significantly decrease further mineral loss [12].

In recent years, several in vivo or in situ studies have focused on the clinical use of nHA, but to this date, no quantitative data synthesis has been published focusing on the efficacy of agents containing (fluoride-free) nHA. Therefore, the aim of this systematic review and meta-analysis was to critically summarize the literature and evaluate the efficacy of fluoride-free nano-hydroxyapatite agents to enhance remineralization or hamper demineralization of enamel caries.

## Materials and methods

### Review design

The study was registered with PROSPERO (CRD42021258368). The Preferred Reporting Items for Systematic Reviews and Meta-Analyses (PRISMA) were adopted throughout the process of the present systematic review [13]. The PICOS model was used to structure the clinical research question by defining the in- and exclusion criteria (Table 1). Thus, the present review aimed at systematically retrieving and analyzing clinical studies (in vivo and in situ) investigating the efficacy of (nano-/micro-) hydroxyapatite (nHA) dentifrice to reduce the initiation of new initial caries lesions or to remineralize initial caries lesions in patients of any age. For this, control treatment could be any other (placebo) treatment or untreated control or standard control (e.g., fluoride dentifrice), and no restrictions with regard to the outcome were defined.

**Table 1:** PICOS schema: population (*P*), intervention (*I*), comparison (*C*), outcomes (*O*), and study design (*S*)

P	-	Participants: patients of any age with initial caries lesions
I	-	Intervention: fluoride-free (micro-/nano-)hydroxyapatite
C	-	Control: any other (placebo) treatment or untreated control or standard control (e.g., fluoride dentifrice)
O	-	Outcome: primary development of caries lesion (initiation and progression/regression), e.g., laser fluorescence, ICDAS score, enamel remineralization rate, mineral loss, and lesion depth
S	-	Studies: randomized controlled clinical (in vivo and in situ) trials (RCTs), prospective controlled clinical trials (CCTs), prospective and retrospective cohort studies, studies with split-mouth, parallel-arm, or crossover designs. The minimum follow-up period had to be 1 month for in vivo studies and 14 days for in situ studies

### Search strategy

Detailed search strategies were developed and appropriately revised for each database, considering the differences in controlled vocabulary and syntax rules by two authors (R. J. W., T. S. C). The search strategies for MEDLINE/PubMed are shown in Table 2. The following electronic databases were searched to find reports of relevant published studies:

- The Cochrane Central Register of Controlled Trials (CENTRAL) (up to April 30, 2021)
- MEDLINE (PubMed) (1946 to April 30, 2021)
- Ovid EMBASE (1947 to April 30, 2021)

**Table 2:** Search strategy as used for PubMed

Search	Query	Results
#1	Remineralization	4216
#2	Demineralization	12.103
#3	Remineralisation	463
#4	Demineralisation	12.113
#5	Cavit*	237.697
#6	Decay	94.937
#7	Carious	7124
#8	Caries	63.006
#9	(((((remineralization) OR (demineralization)) OR (cavit*)) OR (decay)) OR (carious)) OR (caries)	393.322
#10	BioRepair	20
#11	Karex	6
#12	Nano-hydroxyapatite	1.084
#13	Nanohydroxyapatite	1.171
#14	Nano-HA	184
#15	Hydroxyapatite	32.503
#16	Micro-hydroxyapatite	20
#17	Microhydroxyapatite	22
#18	Micro-HA	14
#19	((((((((nano-hydroxyapatite) OR (nanohydroxyapatite)) OR (nano-HA)) OR (micro-hydroxyapatite)) OR (microhydroxyapatite)) OR (micro-HA)) OR (BioRepair)) OR (Karex)) OR (hydroxyapatite)	32.709
#20	Toothpaste	5.795
#21	Dentifrice	7.675
#22	Gel	444.794
**#23**	Varnish	11.738
#24	Solution	846.221
#25	((((toothpaste) OR (dentifrice)) OR (gel)) OR (varnish)) OR (solution)	1.281.280
#26	(((((((((remineralization) OR (demineralization)) OR (remineralisation)) OR (demineralisation)) OR (cavit*)) OR (decay)) OR (carious)) OR (caries)) AND (((((((((nano-hydroxyapatite) OR (nanohydroxyapatite)) OR (nano-HA)) OR (micro-hydroxyapatite)) OR (microhydroxyapatite)) OR (micro-HA)) OR (BioRepair)) OR (Karex)) OR (hydroxyapatite))) AND (((((toothpaste) OR (dentifrice)) OR (gel)) OR (varnish)) OR (solution))	645

No language or time restrictions were applied. Two authors (R. J. W., T. S. C.) independently reviewed the titles and abstracts. The reviewers were not blinded to the identity of the journal names or article authors, their institutions, or the results of their research. A detailed sequence of filtering search results to include relevant articles can be found in the supplementary material. In order to further identify potential articles for inclusion, gray literature was searched in the register of clinical studies hosted by the US National Institutes of Health (www.clinicaltrials.gov), the multidisciplinary European database (www.opengrey.eu), the National Research Register, and ProQuest Dissertation Abstracts and Thesis databases. Agreement concerning study inclusion or data extraction was achieved by consultation and discussion with a third author (T. G. W.). The full text of the selected articles was screened. Cross-referencing was performed to identify further articles to be assessed.

### Data collection

Two authors performed data extraction independently and in duplicate (R. J. W., T. S. C.). The following data was collected in pre-defined excel sheets: author/title/year of study, study affiliation data, study type and setting, design of the study, number/age/gender of patients, intervention applied, inclusion criteria and outcome definitions, outcome assessed with all relevant clinical variables (e.g., laser fluorescence, ICDAS score, enamel remineralization rate, mineral loss, and lesion depth), dropouts, follow-up (maximum follow-up over all groups was used), sources of funding, trial registration, and publishing of the trial’s protocol.

For longitudinal studies and clinical trials presented in different journals or in different publication years, results were presented within one column. Studies without enough data for meta-analyses were kept in the systematic review, but they were excluded from the meta-analyses.

### Data synthesis and grading

Meta-analyses were conducted if studies with similar comparisons reported the same outcomes. For continuous variables, the primary measures of effect between treatment and control groups were the mean differences (MDs) for studies using the same outcome, and for studies using the same construct but different scales, the standardized mean differences (SMDs) were used [14]. For dichotomous outcome data (e.g., surface texture), the primary measures of effect were risk ratios (RRs) and 95% confidence intervals (95% CI) [15].

Statistical heterogeneity was assessed using a *χ*^2^ test and the *I*^2^ statistic [16] using Review Manager (RevMan version 5.4 software, Cochrane Collaboration, Copenhagen, Denmark, 2014). Fixed or random-effect meta-analyses were performed depending on the heterogeneity (*I*^2^ < 35%, fixed effects; *I*^2^ > 35%, random effect) [8, 14]. Risk of bias for interventional, randomized controlled trials (RCTs) was performed independently and in duplicate (G. C., T. G. W.) using the Risk of Bias 2.0 tool [17] and for interventional, non-randomized controlled trials using the ROBINS-I-tool [18]. For in situ studies, these criteria were slightly modified as done previously [19]. Grading of evidence was performed according to the GRADE network levels using GRADE profiler 3.6. [20]. Publication bias was assessed by funnel plots [21].

To avoid unit-of-analysis errors, the guidelines outlined by the Cochrane Collaboration (Chapter 9.3.4.) were followed [22]. Therefore, baseline data were compared with data of a single time point (mostly longest follow-up period).

### Sensitivity analysis

We explored whether or not the analysis of studies stratified by (i) risk of bias or (ii) study design yielded similar or different results. For this, (i) studies at high risk of bias or (ii) studies using split-mouth designs were eliminated in a second/third analysis.

## Results

**Fig. 1:**
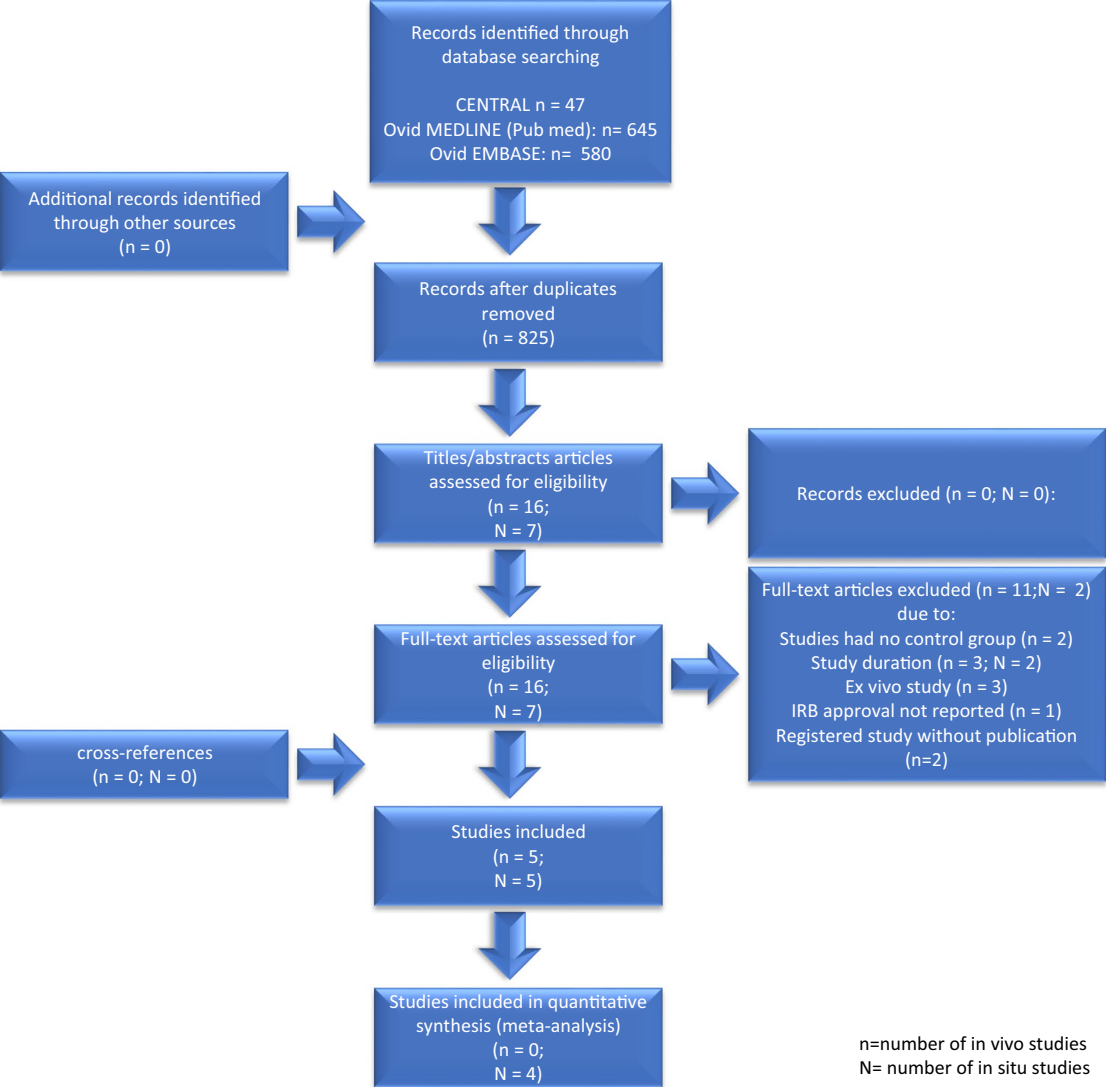
Study flow

A total of 825 studies were initially identified, and after title and abstract screening, 16 in vivo and 7 in situ studies were assessed for eligibility. After the full-text screening, 13 studies were excluded (Fig. 1, online supplementary material Table 1).

### In vivo* studies*

From the 16 studies identified, 11 were excluded because they were either registrations of controlled trials, or because of the lack of a control group, a short study duration of less than 1 month, not reporting an IRB approval, or due to an ex vivo characteristic (Fig. 1). So, 5 studies were included in the review, with a total of 633 teeth with initial caries lesions in 420 patients with an age range of 3–40 years. All studies were randomized controlled studies, three investigating the efficacy of nHA in non-orthodontic patients [23–25] and two investigating the efficacy of nHA in orthodontic patients [26, 27]. A fluoride-free nHA agent was compared to a fluoride-containing pendent in all studies; only one additionally used fluoridated toothpastes in all test groups [23]. Regular close-meshed visits [23–27] including regular oral health instructions [23–27] were planned in all studies. Furthermore, the teeth were professionally cleaned at each recall visit in at least three studies [23, 24, 27]. Two studies did not report if the teeth had been cleaned at all [25, 26]. The reported outcomes were ICDAS score [24, 27], laser fluorescence (mostly DIAGNOdent values) [23, 26], photographic pixels [26], and enamel acid resistance [25]. Interestingly, overall ICDAS scores have been reported to be used in four studies [23, 24, 26, 27], but results were only reported in two. Two studies analyzed the progression of enamel caries lesions [23, 26], one (presumably only) the initiation of new enamel lesion [25], and two studies analyzed the progression of existing enamel lesions and the initiation of new enamel lesion without differentiating between both [24, 27]. Three studies used the tooth level for clinical and statistical evaluation [23, 25, 26], whereas the other two used the tooth level for clinical evaluation and the patient level for statistical evaluation [24, 27]. In two studies, at least one author was identical, and a non-inferiority design was used [24, 27]. Furthermore, three of the five studies were funded by the manufacturer of the tested products [24, 26, 27]. An overview of the main characteristics of the included studies is presented in the online supplementary material Table 2.

Due to the high heterogeneity (including population, control group, outcomes, follow-up periods, high risk of bias, etc.) and statistical designs (e.g., non-inferiority analysis) of the included studies, no meta-analysis was performed. If the study designs created conditions that do not reflect the everyday conditions, the toothpastes were able to remineralize the lesions, but generally, no significant differences were observed between nHA and the fluoridated pendant [23, 24, 26, 27]; only in one case nHA showed a significantly higher effect than the non-fluoridated pendant [25].

### In situ* studies*

From the 7 studies identified, 2 were excluded because of a short study duration. Eventually, 5 studies with 1031 specimens with initial caries lesions or sound surfaces in more than 95 patients were included. All studies were crossover in situ studies, all investigating the efficacy of nHA on initial caries lesions [10, 28–31] and sound surfaces [10, 28, 29, 31]. The outcomes were described using mineral loss and lesion depth using transversal microradiographic images [10, 28, 29, 31] or laser fluorescence (DIAGNOdent values) [30]. Two studies used in situ models which generated overall remineralization conditions [10, 30, 31], one demineralization conditions [28], and one used an in situ model which generated overall remineralization conditions for artificial lesions and overall demineralization conditions for sound surfaces [29]. Two studies were performed in the same department, and at least one author was identical [10, 31], who also cooperated in a series of research projects with employees of the firm producing one of the tested products and who also financed the studies [32]. An overview of the main characteristics of the included studies is presented in the online supplementary material Table 2.

Meta-analyses could be performed for the following comparisons: fluoride-free nHA vs. fluoridated control [10, 28, 31] as well as (fluoridated) nHA vs. nHA-free control [28, 29].

The in situ studies indicated that under demineralization conditions, significantly more demineralization can be observed for nHA than for NaF (mineral loss: MD [95% CI] = 1625 [553, 2697]), and no significant difference can be observed between nHA and fluoride-free control (mineral loss: MD [95% CI] =  − 61 [− 1301, 1179]) (online supplementary material Fig. 1). In contrast, under remineralizing conditions, nHA and NaF show the same remineralizing potential (mineral loss: MD [95% CI] =  − 15 [− 133, 103]), and significantly, more remineralization could be observed for nHA than for negative control (mineral loss: MD [95% CI] = 350 [179, 521]). However, the level of evidence was very low. Furthermore, only two studies were funded by a national grant [28, 33].

### Quality assessment

**Fig. 2:**
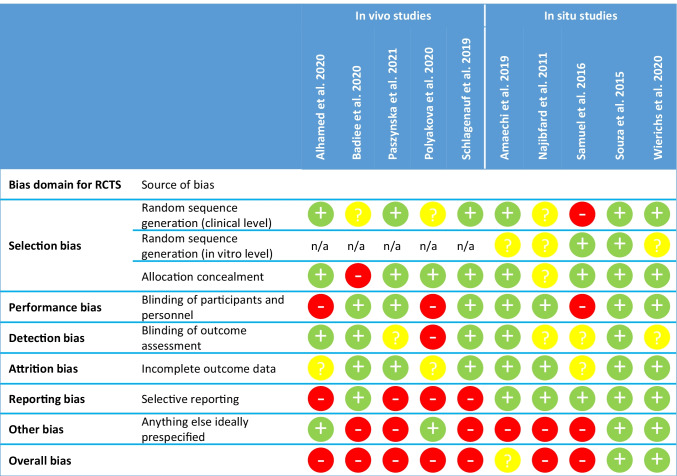
Risk of bias assessment. For interventional, randomized controlled trials (RCTs), the Risk of Bias 2.0 tool was used

Of the 10 trials, quality of 2 was assessed as low [28, 29], 1 as unclear [31], and 7 as high risk of bias [10, 23–27, 30] (Fig. 2). Grading of evidence for meta-analyses showed a very low level of evidence (*data not shown*).

### Sensitivity analysis

When excluding studies at high risk of bias (or studies with different study designs, such as split-mouth design), no meta-analysis was possible. Thus, no sensitivity analysis could be performed.

## Discussion

The efficacy of nHA on buccal or occlusal initial caries lesions as well as on sound enamel surfaces has been critically summarized. Studies using a wide range of outcomes have been extracted. ICDAS score, laser fluorescence, enamel remineralization rate, mineral loss, and lesion depth were the outcomes. The median follow-up period for the included in vivo studies was only 6 months with a range between 6 and 12 months, while for in situ studies, the median was 21 days, ranging between 14 and 28 days. Data from the in vivo studies were not conclusive as study designs resulted in conditions that do not reflect the everyday conditions. Nonetheless, the meta-analysis from the in situ studies suggested that nHA may not hinder enamel demineralization, but it might be a viable option to remineralize enamel caries, albeit only when remineralization conditions are already present.

Remineralization conditions in the mouth can easily be achieved because of saliva. Saliva can provide a natural remineralization. However, to include saliva remineralization in clinical studies, and thus to show clinically more relevant results, study designs must be longer than 21 days [34]. This probably occurred in the in vivo studies, where the median follow-up period was 5.6 months. However, this time frame still seems to be quite short when compared to the advised 3-year follow-up period for direct restorations and the advised 5-year follow-up for indirect restorations [35]. In contrast, the median duration of the in situ studies was 21 days only. Actually, two of the included in situ studies analyzed the effect of nHA for a relatively short period of 14 days [29, 31]. Thus, natural remineralization could not develop its full effect in every in situ study [36]. Consequently, firstly, differences between non-fluoridated and fluoridated groups which would be detectable in the first days might disappear over time if the effect of natural remineralization is larger than the effect of the tested ingredients, and secondly, in fluoridated test groups, the enhanced natural remineralization might not be observed in the first days of a study.

One of the most major limitations of the present review is the research designs of the in vivo studies. Regular close-meshed visits (between 2 weekly and 3 monthly) [23–25, 27], regular oral health instructions [24, 27], brushing diary [24] and supervised brushing [24], and regular application of chlorhexidine [27] create conditions that do not reflect the everyday conditions of the average patient. Starting from this premise, the positive outcomes noted in some studies are not totally due to the nHA but rather (also partly) related to the changes to the patient’s previous treatment and to the “positive” conditions of the clinical study itself [37, 38]. These conditions might vary according to study designs, where in retrospective (practice-based) studies, the data collection with regard to exposures, confounders, and endpoint might not be very accurate, but also it cannot be artificially influenced [39]. While in prospective clinical studies, optimized management (professional dental prophylaxis and/or thorough oral health instructions) before beginning the randomized phase of treatment might already lead to positive outcomes [37]. Also, the patient or their parents/caregivers in case of children are aware that they are being observed, graded, or measured, which results in changes in their behavior (Hawthorne effect) [38], and this can also lead to positive (though transient) results.

Consequently, the results of the in vivo studies can only be viewed considering these conditions used in the studies, and they cannot be generalized. This underlines the concept that regular dental checkups are the first choice to manage caries lesions, because the patients can be remotivated to perform oral hygiene properly [40], highlighting also the need for more in vivo studies to evaluate the clinical effect of nHA.

As written above, nHA only showed a positive result in enamel caries remineralization when the remineralizing conditions were already present, and this again highlights the Hawthorne effect as explained previously, and that patient motivation during regular checkups might help maintain the remineralizing conditions necessary for the positive effect of nHA.

The scientific evidence might be additionally limited by the sponsorship and/or authorship of the studies. Three in vivo and two in situ studies were funded either by the manufacturer [24, 26, 27] or one author [10, 24, 31] meanwhile cooperates with employees of the manufacturer of the tested products in a series of research projects which are financially supported by the manufacturer. All these factors had an impact on the risk of bias analysis and evidence grading.

Although a positive effect of nHA has not yet been proven in the in vivo studies under everyday conditions and with long-term follow-ups, the results of the in situ studies might have some clinical relevance for the future. In daily clinical practice, dentists are often confronted by patients who, despite numerous attempts to elucidate the blatant evidence regarding fluoride, wholeheartedly resist using fluoridated products. If the remineralizing effects of nHA (that were observed only in the in situ studies) would be also proven to be valid in in vivo situations, then nHA could be a viable option for such patients who also present active caries lesions, as long as they are involved in optimized management and are remotivated to perform oral hygiene properly.

In conclusion, the low number of clinical studies with relatively short follow-up periods, high risks of bias, limiting grade of evidence, and study conditions that do not reflect the everyday conditions does not allow for stating an evidence on the efficacy of nHA to enhance remineralization or hamper demineralization of enamel caries.

## Supplementary Information

Below is the link to the electronic supplementary material.

Supplementary file1 (DOCX 54 KB)

Supplementary file2 (PPTX 84 KB)

## Data Availability

All data generated or analyzed during this study are included in this article and/or its supplementary material files. Further inquiries can be directed to the corresponding author.
