# Supplementary material for: Efficacy of nano-hydroxyapatite on caries prevention—a systematic review and meta-analysis
Source: Clin Oral Investig. 2022 Feb 1;26(4):3373–81. doi: 10.1007/s00784-022-04390-4 (PMC8979882; doi:10.1007/s00784-022-04390-4)
Supplement: Supplementary file 1 — Supplementary file1 (DOCX 54 KB) [file 784_2022_4390_MOESM1_ESM.docx]

**Caries preventive efficacy of nano-hydroxyapatite - a meta-analysis**

**online supplementary material**

Sequence of filtering search results

The sequence of filtering search results in order to include relevant articles in the review was as follows:

1. articles written in German, Portuguese, French, Italian or English,
2. articles written in languages of which the authors were able to get a vague idea of the content (Latin-based languages) or those having only a translated (German, Portuguese, French, Italian, English or Russian) abstract,
3. articles written in languages the authors were not able to understand at all.

After deciding on the German, Portuguese, French, Italian and English articles the authors read through the category 2) articles realizing that only one of them fulfilled inclusion criteria. Of course, for all category 3) articles (presumably 0 studies), the authors were not able to make a decision based on their content.

**online supplementary material table 1**: Excluded studies

| Author (Year) | Study design | Reason for exclusion |
| --- | --- | --- |
| [1] | in vivo | Ex vivo study |
| [2] | in vivo | Study duration |
| [3] | in vivo | No control group |
| [4] | in situ | Study duration |
| [5] | in vivo | Ex vivo study |
| [6] | in vivo | No control group, lesions totally remineralized within 3 days |
| [7] | in vivo | Study duration |
| [8] | in vivo | Study duration |
| [9] | in vivo | IRB approval not reported |
| [10] | in vivo | Ex vivo study, study duration |
| [11] | in situ | Non-cariogenic study |

**online supplementary material Figure 2:** Quantitative meta-analyses for outcomes mineral loss. The primary measures of effect between treatment and control groups were the mean differences (MD) for studies using the same outcome and standardized mean differences (SMD) for studies using the same construct but different scales.). Forest plots, heterogeneity parameter (I2) as well as overall statistics (Z, P) are given.

**Appendix table 2** (part 1): Detailed summary of included in vivo and in situ studies

| **First author** | **Type of lesion and tooth** | **Type of Intervention(s)** | **Mean age of patients [mean (SD), range] [years]** | **Study design** | **Follow-up time(s) [months]** | **Number of patients (M, F) at the beginning, number of teeth (per group)** | **Number of patients (M, F) at the end,  number of teeth (per group)** |
| --- | --- | --- | --- | --- | --- | --- | --- |
| **In vivo** |  |  |  |  |  |  |  |
| **Alhamed et al. 2020** | pits and fissure initial caries, smooth surface initial caries non-orthodontic patients ICDAS score: not reported | **gel/varnish** 1. Tricalcium phosphate + F toothpaste (1450 ppm F) 2. Fluoride varnish (22,600 ppm F) + F toothpaste (1450 ppm F) 3. nHA gel + F toothpaste (1450 ppm F)  application frequency: once every week | 20-40 | RCT, parallel-arm examiner-blinded | T0:0 0.25 0.5 0.75 T1: 1 | not reported  90 teeth (30, 30, 30) | Not reported teeth not reported |
| **Badiee et al. 2020** | White spot lesions in orthodontic patients ICDAS score: 2-3 | **toothpaste** 1. nHA  2. F (“ordinary F containing toothpaste”; ppm F not being reported)  brushing instruction at every appointment (1-, 3-, 6-months) | 10-35 | RCT, parallel-arm double-blinded | T0: 0 1 3 T1: 6 | 50 (17, 33)  173 teeth (77/96) | 50 (17, 33)  173 teeth (77/96) |
| **Paszynska et al. 2021** | primary molars in a very high cariogenic population ICDAS score: 0-2 | **toothpaste** 1. nHA ( 2. F (500 ppm F)  Oral Health instruction and supervised brushing every 84 days supervised brushing 2x/d (incl. brushing diary) | nHA: 4.9 F: 5.2 3-7 | RCT, parallel-arm double-blinded | -0-3 T0: 0 2.8 5.6 8.4 T1: 11.2 | 214 (n/a, n/a)  Teeth not reported | 177 (76/100)  Teeth not reported |
| **Polyakova et al. 2020** | presumably sound enamel non-orthodontic patients | **toothpaste** 1. test: calcium nitrate (brushite) (two bottles) [Ca] 2. "positive control": nHA containing (one bottle) 3. Negative control: fluoride-free, nHA-free (one bottle) | 20-25 | RCT, parallel-arm double-blinded (patient could not have been blinded (see number of bottles per group) | T0: 0 0.5+subseqent daily T1: 1+ subsequent daily | 63 (n/a, n/a)  Teeth not reported | 60 (25, 35)  Teeth not reported |
| **Schlagenhauf et al. 2019** | ICDAS score: 0-2 highly caries-active orthodontic patients | **toothpaste** 1. nHA 2. NaF (1400 ppm F)  CHX was applied every 4 weeks + professional tooth cleaning | nHA: 13.4±1.7 NaF: 13.4±1.8 11-25 | RCT, parallel-arm double-blinded | -0.9 T0: 0 0.9 1.8 2.8 3.7 T1: 5.6 | 150 (76/ 74)  Teeth not reported | 133 (64/69)  Teeth not reported |
| **First author** | Type of lesion and tooth | **Type of Intervention(s)** | Mean age of patients [mean (SD), range] [years] | Study design | Follow-up time(s) [days] | Number of patients (M, F) at the beginning, number of teeth (per group) | Number of patients (M, F) at the end,  number of teeth (per group) |
| **In situ** |  |  |  |  |  |  |  |
| **Amaechi et al. 2019** | sound and artificial lesion human enamel | **toothpaste** 1. nHA: 10%  2 NaF (500 ppm F) | 39.5 (15.0) 18-60 | Randomized cross-over controlled study | 14 | 32 (n/a, n/a)  64 samples (32, 32) | 30 (11, 19)  60 samples (30, 30) |
| **Najibfard et al. 2011** | sound and artificial lesion human enamel | **toothpaste** carious lesions 1. nHA: 5% 2. nHA: 10%  3. NaF: 1100 ppm F toothpaste  sound surfaces 4. nHA: 10% | 37.8 (7.9) 21-40 | Randomized cross-over controlled study | 28 | 30 (12, 18)  30 per subgroup | 30 (12, 18)  30 per subgroup |
| **Samuel et al. 2016** | artificial lesion  human enamel | **water + paste** 1. ozone+nHA:ozonated water and 10 % nano-hydroxyapatite 2. nHA: 10 % nano-hydroxyapatite  3. Negative control (saliva) |  | Randomized cross-over controlled study | 21 | n/a | n/a  10 samples overall or per group |
| **Souza et al. 2015** | sound and artificial lesion human enamel sound and artificial lesion human dentin human teeth | **pastes** 1. nHA+NaF: 10% HA + 0.2% NaF  2. CPP-ACP+NaF: casein phosphopeptide-amorphous calcium phosphate + 0.2% NaF  3. NaF: 0.2% NaF 4. Negative control (0ppm F) | 19-28 | Randomized cross-over controlled study | 14 | 13 (2, 11)  26 sound and  26 pre-demineralized specimen per group per substrate (enamel/dentin) | 13 (2/11)  26 sound and  26 pre-demineralized specimen per group per substrate (enamel/dentin) |
| **Wierichs et al. 2020** | sound and demineralized enamel sound and demineralized dentin bovine teeth | **toothpaste**  1. nHA: 2. Negative control: (0 ppm F)  3. Standard control (1100 ppm F)  4. Positive control (5000 ppm F) | 20-56 | Randomized cross-over controlled study | 28 | 20 (5, 15) 120 (40 per subgroup) | 20 (5, 15) 30-38 depending on the subgroup |

**Appendix table 2** (part 2): Detailed summary of included in vivo and in situ studies

| **First author** | **Measure of outcome** | **outcome: ICDAS** | **outcome: photographic pixel** | **outcome: DIAGNOdent/Cariescan [mean (SD)]** | **Country** | **Cave:** |
| --- | --- | --- | --- | --- | --- | --- |
| **In vivo** |  |  |  |  |  |  |
| **Alhamed et al. 2020** | DIAGNOdent ICDAS (results not presented) | - | - | T0 TCP: 16.00±3.714 FV: 16.90±3.633 nHA: 15.50±3.58  T5w TCP: 10.37±3.37 FV: 10.60±2.74 nHA: 7.80 ±3.13 | Umm Al-Qura University, Saudi Arabia | - |
| **Badieeet al. 2020** | DIAGNOdent photographs plaque index ICDAS (results not presented) | **-** | T0 Difference nHA-F: 268 pixel (p=0,736)  T6 Difference nHA-F: 82 pixel (p=n/a) | T0 Difference nHA-F:0.3 (p=0.878)  T6 Difference nHA-F: 0.8 (p=n/a) | Shahid Beheshti University of Medical Sciences, Tehran,  Iran | ICDAS 2-3 included |
| **Paszynska et al. 2021** | ICDAS (new caries lesions or progression of a lesion) modified Gingival Index Plaque Control Record | T0-T12 nHA: increase in 64 teeth (total 88) F: increase in 66 teeth (total 89) Risk difference is also presented |  | - | Poznan and Bialystok, Poland | clinical evaluation on tooth level  statistical evaluation on patient level |
| **Polyakova et al. 2020** | simplified oral hygiene index (OHI-S),  enamel acid resistance test enamel remineralization rate  Shiff test | Enamel remineralization rate [mean (SD)] T0 Ca: 2.9±0.3 nHA: 3.250.44 NC: 2.95±0.51  T1 Ca: 1.8±0.61 nHA: 2.5±0.5 NC: 2.96±0.53 T0-T1 Ca vs. NC p>0.001 nHA vs. NC p=0.113 | Enamel acid resistance  T0 [mean (SD)] Ca: 4.35±0.59 nHA: 4.5±0.69 NC: 4.35±0.67  T1 Ca: 3.15±0.58 nHA: 3.0±0.65 NC: 4.3±0.73 T0-T1 Ca vs. NC p>0.001 nHA vs. NC p=0.00074 | - | Sechenov University, Moscow,  Russia | - |
| **Schlagenhauf et al. 2019** | Number of lesion developments ICDAS≥code 2 Plaque index gingival index | no baseline values are given |  | - | Würzburg, Munich, Frankfurt, Dresden and Regensburg, Germany | clinical evaluation on tooth level  statistical evaluation on patient level |
| **First author** | **Measure of outcome** | **outcome: mineral loss** | **outcome: lesion depth pixel** | **outcome: DIAGNOdent/Cariescan [mean (SD)]** | **Country** | **model** |
| **In situ** |  |  |  |  |  |  |
| **Amaechi et al. 2019** | microradiography (mineral loss and lesion depth) | T0 nHA: 2358±455 NaF: 2379±593  T14 nHA: 1014±274 NaF: 1009±392  T0-T14 (95%CI) nHA: 1344 (1120-1568) NaF: 1370 (117-1622) | T0 nHA: 92,.9±17.2 NaF: 91.9±17.9  T14 nHA: 67.1±11.8 NaF: 65.5±13.6  T0-T14 (95%CI) nHA: 25.8 (19.8-31.8) NaF: 26.44 (21.2-31.7) | - | University of Texas Health San Antonio, Texas USA | net-remineralisation |
| **Najibfard et al. 2011** | microradiography (mineral loss) | T0 nHA 5%: 1295±418 nHA 10%: 1387±351 NaF: 1268±421  T28 nHA 5%: 860±351 nHA 10%: 998±319 NaF: 864±272  T0-T28 (95%CI) nHA5%: 434 (328-541) nHA10% : 389 (325-452) NaF: 404 (298-507)  sound surfaces:  no demineralization in a net-remin in situ model | T0 nHA 5%: 73.8±13.9 nHA 10%: 74.8±13.1 NaF: 72.5±15.1  T28 nHA 5%: 62.7±11.8 nHA 10%: 66.8±11.7 NaF: 64.6±15.0  T0-T28 (95%CI) nHA5%: 11.1 (7.2-14.9) nHA10% : 8.0 (5.1-10.9) NaF: 7.9 (3.6-12.2) | **-** | University of Texas Health San Antonio, Texas USA | net-remineralisation |
| **Samuel et al. 2016** | DIAGNOdent Polarized light microscopic analysis | - | **-** | T0 ozone+ nHA: 20.5±3.1 nHA: 19.9±2.8 NC: 20.8±3.2  T21 ozone+ nHA: 8.1±1.5 nHA: 12.0±2.3 NC: 17.0±2.6 | Thai Moogambigai Dental College and Hospital Chennai,  India | net-remineralisation |
| **Souza et al. 2015** | transversal microradiography | T0-T21 (dentin sound) nHA+F:-780±212 CPP-ACP:-876±268 NaF:-900±236 NC:-1188±503  T0-T21 (dentin artificial lesion) nHA+F:910±329 CPP-ACP:964±446 NaF:902±607 NC:338±408  T0-T21 (enamel sound) nHA+F:-1001±250 CPP-ACP:-884±432 NaF:-985±313 NC:-1370±988  T0-T21 (enamel artificial lesion) nHA+F:550±405 CPP-ACP:371±231 NaF:556±264 NC:200±187 | T0-T21 (dentin sound) nHA+F:-99±26 CPP-ACP:-96±30 NaF:-96±26 NC:-143±28  T0-T21 (dentin artificial lesion) nHA+F:22±18 CPP-ACP:20±19 NaF:19±34 NC:5±13  T0-T21 (enamel sound) nHA+F:-45±15 CPP-ACP:-61±26 NaF:52±21 NC:-57±31  T0-T21 (enamel artificial lesion) nHA+F: 24±14 CPP-ACP:9±13 NaF:13±8 NC:8±6 | **-** | Bauru School of Dentistry, University of São Paulo, Bauru ,  Brazil | net-remineralisation for demineralized specimens  net-demineralisation for sound surfaces |
| **Wierichs et al. 2020** | transversal microradiography | T0-T28 enamel (95%CI) nHA: -1787 (-2725;-850) NC: -1848 (-2737;-959) NaF1100: -162 (-768;443) NaF5000: 1076 (257;1894)  T0-T28 dentin lowly nHA: -1002 (-1855;-149) NC: -560 (-1164;43) NaF1100: 728 (310;1147) NaF5000:1146 (458;1833)  T0-T28 dentin highly nHA: 13 (-741;767) NC: -220 (-869;429) NaF1100: 1515 (963;2067) NaF5000: 3117 (2660;3574)  baseline and effect values were also given | - | **-** | Aachen, Germany | net-demineralisation |

**References**

1. Bossù M, Saccucci M, Salucci A, Di Giorgio G, Bruni E, Uccelletti D, Sarto MS, Familiari G, Relucenti M and Polimeni A (2019) Enamel remineralization and repair results of Biomimetic Hydroxyapatite toothpaste on deciduous teeth: an effective option to fluoride toothpaste. J Nanobiotechnology 17:17. doi: 10.1186/s12951-019-0454-6

2. Ebrahimi M, Mehrabkhani M, Ahrari F, Parisay I and Jahantigh M (2017) The effects of three remineralizing agents on regression of white spot lesions in children: A two-week, single-blind, randomized clinical trial. J Clin Exp Dent 9:e641-e648. doi: 10.4317/jced.53582

3. Grocholewicz K, Matkowska-Cichocka G, Makowiecki P, Droździk A, Ey-Chmielewska H, Dziewulska A, Tomasik M, Trybek G and Janiszewska-Olszowska J (2020) Effect of nano-hydroxyapatite and ozone on approximal initial caries: a randomized clinical trial. Sci Rep 10:11192. doi: 10.1038/s41598-020-67885-8

4. Hannig C, Basche S, Burghardt T, Al-Ahmad A and Hannig M (2013) Influence of a mouthwash containing hydroxyapatite microclusters on bacterial adherence in situ. Clin Oral Investig 17:805-14. doi: 10.1007/s00784-012-0781-6

5. Lelli M, Putignano A, Marchetti M, Foltran I, Mangani F, Procaccini M, Roveri N and Orsini G (2014) Remineralization and repair of enamel surface by biomimetic Zn-carbonate hydroxyapatite containing toothpaste: a comparative in vivo study. Front Physiol 5:333. doi: 10.3389/fphys.2014.00333

6. Makeeva IM, Polyakova MA, Avdeenko OE, Paramonov YO, Kondrat'ev SA and Pilyagina AA (2016) [Effect of long term application of toothpaste Apadent Total Care Medical nano-hydroxyapatite]. Stomatologiia (Mosk) 95:34-36. doi: 10.17116/stomat201695434-36

7. Makeeva IM, Polyakova MA, Avdeenko OE, Paramonov YO, Kondrat'ev SA and Pilyagina AA (2016) Optimization of dental caries prevention. Stomatologiia 95:34-36.

8. Makeeva IM, Polyakova MA, Doroshina VY, Sokhova IA, Arakelyan MG and Makeeva MK (2018) [Efficiency of paste and suspension with nano-hydroxyapatite on the sensitivity of teeth with gingival recession]. Stomatologiia (Mosk) 97:23-27. doi: 10.17116/stomat20189704123

9. Makeeva IM, Polyakova MA, Doroshina VY, Turkina AY, Babina KS and Arakelyan MG (2018) [Comparative effectiveness of therapeutic toothpastes with fluoride and hydroxyapatite]. Stomatologiia (Mosk) 97:34-40. doi: 10.17116/stomat20189705134

10. Sudradjat H, Meyer F, Loza K, Epple M and Enax J (2020) In Vivo Effects of a Hydroxyapatite-Based Oral Care Gel on the Calcium and Phosphorus Levels of Dental Plaque. Eur J Dent 14:206-211. doi: 10.1055/s-0040-1708456

11. Wu X, Zhao X, Li Y, Yang T, Yan X and Wang K (2015) In situ synthesis carbonated hydroxyapatite layers on enamel slices with acidic amino acids by a novel two-step method. Mater Sci Eng C Mater Biol Appl 54:150-7. doi: 10.1016/j.msec.2015.05.006
