# Supplementary material for: Efficacy of nano-hydroxyapatite on caries prevention—a systematic review and meta-analysis
Source: Clin Oral Investig. 2022 Feb 1;26(4):3373–81. doi: 10.1007/s00784-022-04390-4 (PMC8979882; doi:10.1007/s00784-022-04390-4)
Supplement: Supplementary file 2 — Supplementary file2 (PPTX 84 KB) [file 784_2022_4390_MOESM2_ESM.pptx]

## Slide 1
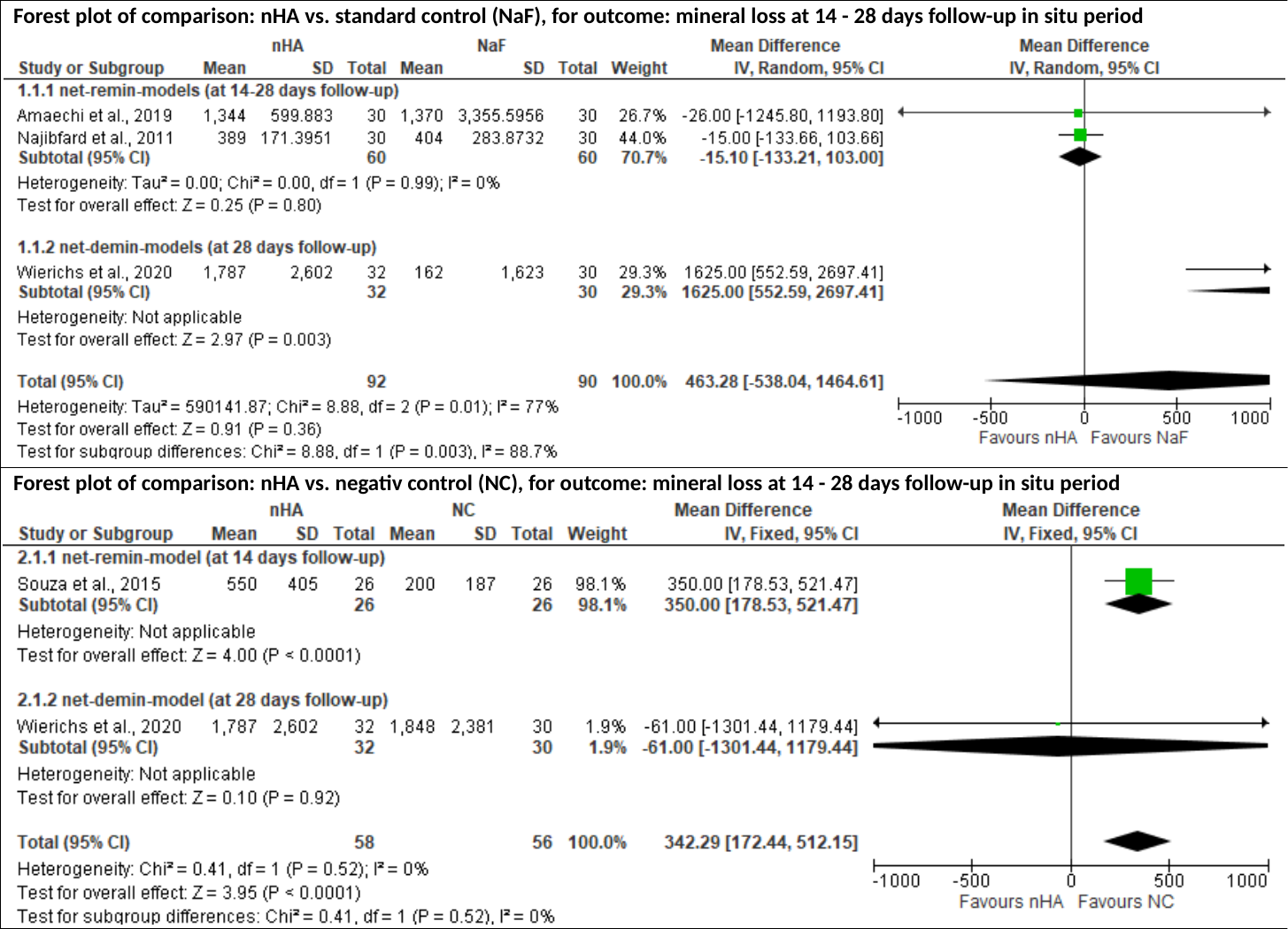

| Forest plot of comparison: nHA vs. standard control (NaF), for outcome: mineral loss at 14 - 28 days follow-up in situ period |
| --- |
| Forest plot of comparison: nHA vs. negativ control (NC), for outcome: mineral loss at 14 - 28 days follow-up in situ period |
| --- |
